# An Analysis Dictionary Learning Algorithm under a Noisy Data Model with Orthogonality Constraint

**DOI:** 10.1155/2014/852978

**Published:** 2014-07-13

**Authors:** Ye Zhang, Tenglong Yu, Wenwu Wang

**Affiliations:** ^1^Department of Electronic Information Engineering, Nanchang University, Nanchang 330031, China; ^2^Centre for Vision, Speech and Signal Processing, University of Surrey, Guildford GU2 7XH, UK

## Abstract

Two common problems are often encountered in analysis dictionary learning (ADL) algorithms. The first one is that the original clean signals for learning the dictionary are assumed to be known, which otherwise need to be estimated from noisy measurements. This, however, renders a computationally slow optimization process and potentially unreliable estimation (if the noise level is high), as represented by the Analysis K-SVD (AK-SVD) algorithm. The other problem is the trivial solution to the dictionary, for example, the null dictionary matrix that may be given by a dictionary learning algorithm, as discussed in the learning overcomplete sparsifying transform (LOST) algorithm. Here we propose a novel optimization model and an iterative algorithm to learn the analysis dictionary, where we directly employ the observed data to compute the approximate analysis sparse representation of the original signals (leading to a fast optimization procedure) and enforce an orthogonality constraint on the optimization criterion to avoid the trivial solutions. Experiments demonstrate the competitive performance of the proposed algorithm as compared with three baselines, namely, the AK-SVD, LOST, and NAAOLA algorithms.

## 1. Introduction

Sparse signal representation has been the focus of much recent research in signal processing fields such as image denoising, compression, and source separation. Sparse representation is often established on the synthesis model [1–3]. Considering a signal **x** ∈ *R* ^*M*^, the synthesis sparse representation of **x** can be described as

(1) $$\begin{array}{c} x=Da\;\text{with}\;\;{||a||}_{0}=k, \end{array}$$

where ||·||_0_ represents the *l*_0_ pseudonorm, defined as the number of nonzero elements of a vector. **D** ∈ *R* ^*M*×*N*^ is a possibly overcomplete dictionary (*N* ≥ *M*), and **a** ∈ *R* ^*N*^, containing the coding coefficients, is assumed to be sparse with *k* ≪ *N*.

Recently, an alternative form of sparse representation model called analysis model was proposed in [4–14]. In this model, an overcomplete analysis dictionary or operator Ω ∈ *R* ^*P*×*M*^ (*P* ≥ *M*) is sought to transform **x** ∈ *R* ^*M*^ to a high dimensional space; that is,

(2) $$\begin{array}{c} \Omega x=z\;\text{with}\;\;{||z||}_{0}=P-l, \end{array}$$

where **z** ∈ *R* ^*P*^ is called the analysis representation of **x** and assumed to be sparse and *l* is cosparsity of the analysis model, which is the number of zeros in the vector **z**.

Both models can be used to reconstruct an unknown signal **x** from the corrupted measurement **y** ∈ *R* ^*M*^; that is,

(3) $$\begin{array}{c} y=x+v, \end{array}$$

where **v** ∈ *R* ^*M*^ is a Gaussian noise vector. This is an ill-posed linear inverse problem, which has been studied extensively [15, 16]. Using the synthesis model, the original signal **x** can be estimated by solving

(4) $$\begin{array}{c} \hat{a}=\underset{a}{\arg\min\;}{||a||}_{0}\;\text{subject}\;\text{to}\;\;{||y-Da||}_{2}^{2}\leq \varepsilon \end{array}$$

and then calculated as $\hat{x}=D\hat{a}$, where *ε* denotes the noise floor. It is now well known [6] that the original signal can also be recovered by solving the following analysis model based optimization problem:

(5) $$\begin{array}{c} \hat{x}=\arg\;\min\;{||\Omega x||}_{0}\;\text{subject}\;\text{to}\;\;{||y-x||}_{2}^{2}\leq \varepsilon . \end{array}$$

For the squared and invertible dictionary, the synthesis and the analysis models are identical with **D** = Ω^−1^ [9]. However, the two models depart if we concentrate on the redundant case (*P* > *M* and *N* > *M*). In the analysis model, the signal **x** is described by the zero elements of **z**, that is, zero entries in **z** that define a subspace which the signal **x** belongs to, as opposed to the few nonzero entries of **a** in the synthesis model.

The performance of both the synthesis and analysis models hinges on the representation of the signals with an appropriately chosen dictionary. In the past decade, a great deal of effort has been dedicated to learning the dictionary for the synthesis model; however, the ADL problem has received less attention with only a few algorithms proposed recently [5, 6, 10, 17–19]. In these works, the dictionary Ω is often learned from the observed signals **Y** = [**y**_1_, **y**_2_,…, **y**_*K*_] ∈ *R* ^*M*×*K*^ measured in the presence of additive noise; that is,

(6) $$\begin{array}{c} Y=X+V, \end{array}$$

where **X** = [**x**_1_, **x**_2_,…, **x**_*K*_] ∈ *R* ^*M*×*K*^ contains the original signals, **V** = [**v**_1_, **v**_2_,…, **v**_*K*_] ∈ *R* ^*M*×*K*^ is a Gaussian noise matrix, and *K* is the number of signals.

Exploiting the fact that a row of the dictionary Ω is orthogonal to a subset of training signals **X**, a sequential minimal eigenvalue based ADL algorithm is proposed in [5]. Once the subset is found, the corresponding row of the dictionary can be updated with the eigenvector associated with the smallest eigenvalue of the autocorrelation matrix of these signals. However, as *P* increases, so does the computational cost of the method. In [6–8], an *l*_1_-norm penalty function is applied to Ω**x**, and a projected subgradient algorithm (called NAAOLA) is proposed for analysis operator learning. These works employ a uniformly normalized tight frame as a constraint on the dictionary to avoid the trivial solution (e.g., Ω = 0), which however limits the range of possible Ω to be learned. In [10], the Analysis K-SVD (AK-SVD) algorithm is proposed for ADL. By keeping Ω fixed, the optimal backward greedy algorithm (OBG) is employed to estimate a submatrix of Ω whose rows are then used to determine a submatrix of **Y** and the eigenvector associated with the smallest eigenvalue of this submatrix is then used to update Ω. A generalized analysis model, that is, the transform model, is proposed in the learning sparsifying transform (LST) algorithm [11]. Unlike the analysis model (2), the sparse representation of a signal in the LST model is not constrained to lie in the range space of Ω. Such a generalization allows the transform model to accommodate a wider class of signals. A closed-form solution to the LST problem is also developed in [13]. The LST algorithm [11] is further extended to the overcomplete case, leading to the LOST algorithm in [12]. The transform K-SVD algorithm proposed recently in [14] is essentially a combination of the ideas in the LOST and AK-SVD algorithms.

There are two problems that have been observed or already studied in some of the ADL algorithms discussed above. The first one is associated with the computational cost in the optimization process of the ADL algorithms. For example, in the AK-SVD algorithm, **X** needs to be estimated before learning the dictionary, and, as a result, the optimization process becomes computationally demanding. The second one is associated with the trivial solutions to the dictionary, which may lead to spurious sparse representations. To eliminate such trivial solutions, extra constraints are required, such as the full-rank constraint on Ω employed in [11–13] and the mutual coherence constraint on the rows of Ω in [12, 14].

In this paper, we propose a new optimization model and algorithm, attempting to provide alternative solutions to the two potential problems mentioned above. More specifically, similar in spirit to our recent work [17], we directly use the observed data to compute the approximate analysis sparse representation of the original signals, without having to preestimate **X** for learning the dictionary. This leads to a computationally very efficient algorithm. Moreover, different from the LOST algorithm, we enforce an orthogonality constraint on Ω, which, as shown later, has led to an improved learning performance.

The paper is organized as follows. In Section 2, we discuss the novel ADL model and algorithm. In Section 3, we show some experimental results, before concluding the paper in Section 4.

## 2. The Proposed ADL Algorithm

In some algorithms discussed above such as [10], the optimization criterion is based on **X**. In practice, however, **X** is unknown and required to be estimated from **Y**. This unfortunately results in a computationally very slow optimization process as shown in Section 3.1. To address this issue, we introduce a new model and algorithm for ADL, where, similar to [17], we use ||Ω**Y**||_1_ as an approximation to ||Ω**X**||_1_, which is further replaced by ||**Z**||_1_ and then used as a new constraint for the reconstruction term **Z** = Ω**Y**. This leads to the following model of ADL:

(7) $$\begin{array}{c} \min\left(\frac{1}{2}{||Z-\Omega Y||}_{F}^{2}+\lambda {||Z||}_{1}\right), \end{array}$$

where *λ* > 0 is a regularization parameter. According to the analysis model (2), **Z** would be an analysis coefficient matrix only if its columns lie in the range space of the analysis dictionary Ω. However, **Z**  =  Ω**Y** is not explicitly enforced in (7), and hence it is called the transform coefficient matrix as in [19]. Using (7), the analysis dictionary Ω is learned from **Y** without having to preestimate **X** as opposed to the AK-SVD algorithm, so the computational effort for estimating **X** from **Y** is exempted.

With the model (7), however, there is a trivial solution; that is, Ω = 0, **Z** = 0. To avoid such a solution, additional constraints on Ω are required. In [12], a full column rank constraint has been imposed on Ω through the use of a negative log determinant of Ω^*T*^Ω. However, the inverse of Ω^*T*^Ω needs to be computed in the conjugate gradient method in [12], and the inverse of Ω^*T*^Ω may affect the stability of the numerical computation when the initial of Ω^*T*^Ω is ill conditioned. To address this problem, in our work, a function defined on Ω^*T*^Ω = **I** is employed as a constraint term which enforces Ω to be a full column rank matrix, based on the fact that the ranks of Ω and its corresponding Gram matrix are equal. This leads to the following new optimization criterion:

(8) $$\begin{array}{c} \min\left(\frac{1}{2}{||Z-\Omega Y||}_{F}^{2}+\lambda {||Z||}_{1}\right)\;\text{subject}\;\text{to}\;\Omega^{T}\Omega =I. \end{array}$$

Using a Lagrangian multiplier *γ*, the optimization problem can be reformulated as

(9) $$\begin{array}{c} \min\left(\frac{1}{2}{||Z-\Omega Y||}_{F}^{2}+\lambda {||Z||}_{1}+\frac{\gamma }{4}{||\Omega^{T}\Omega -I||}_{F}^{2}\right). \end{array}$$

It is worth noting that our proposed objective function (9) is essentially different from the one used in [7]. In [7], the objective function is defined as follows:

(10) $$\begin{array}{c} \min\left({||\Omega X||}_{1}+\frac{\gamma }{4}{||\Omega^{T}\Omega -I||}_{F}^{2}+\frac{\lambda }{4}\underset{i}{\sum }{||w_{i}^{T}w_{i}-\frac{M}{P}||}_{2}^{2}\right), \end{array}$$

where **w**_*i*_ is the *i*th row of Ω. With (10), the analysis dictionary is learned from the clean signal **X** and the sparse constraint is enforced on Ω**X**. In our proposed ADL algorithm, however, the analysis dictionary is directly learned from the noisy observed signal **Y** and thus can tolerate some sparsification errors as in [11]. The results of the experiments demonstrate that our proposed approach is more robust.

Note also that when **Z** is sparse, minimizing ||Ω**Y**||_1_ can be obtained by the minimization of ||**Z**−Ω**Y**||_*F*_^2^, subject to the sparsity constraint. However, both **Z** and Ω are unknown. To solve the problem, we propose an iterative method to alternatively update the estimation of **Z** and Ω.

### 2.1. Estimating **Z**

Given the dictionary Ω, we first consider the optimization of **Z** only. In this case, the objective function (9) can be modified as

(11) $$\begin{array}{c} \hat{Z}=\underset{Z}{\arg\;\min}\left(\frac{1}{2}{||Z-\Omega Y||}_{F}^{2}+\lambda {||Z||}_{1}\right). \end{array}$$

The first-order optimality condition of **Z** implies that

(12) $$\begin{array}{c} Z-\Omega Y+\lambda \mathrm{sign}\left(Z\right)=0. \end{array}$$

Therefore, we have

(13) $$\begin{array}{c} {\hat{Z}}_{ij}=\{\begin{array}{cc} {\left(\Omega Y\right)}_{ij}-\lambda , & {\left(\Omega Y\right)}_{ij}>\lambda ;\\ {\left(\Omega Y\right)}_{ij}+\lambda , & {\left(\Omega Y\right)}_{ij}<-\lambda ;\\ 0 & \text{otherwise}, \end{array} \end{array}$$

where *i* = 1,2,…, *P* and *j* = 1,2,…, *K* are the indices of the matrix elements. It is well known that the above solution for **Z** is called soft thresholding. Indeed, the sparsity constraint ||**Z**||_1_ is only an approximation to ||**Z**||_0_. In order to promote sparsity and to improve the approximation, one could instead use the hard thresholding method [20] as an alternative, that is, setting the smallest components of the vectors to be zeros while retaining the others:

(14) $$\begin{array}{c} {\hat{Z}}_{ij}=\{\begin{array}{cc} {\left(\Omega Y\right)}_{ij}, & \left|{\left(\Omega Y\right)}_{ij}\right|>\lambda ;\\ 0, & \text{otherwise}. \end{array} \end{array}$$

As such, the solution obtained using the constraint ||**Z**||_1_ will be closer to that using ||**Z**||_0_.

### 2.2. Dictionary Learning

For a given **Z**, the objective function (9) is nonconvex with respect to Ω, similar to the objective function (10); that is,

(15) Ω^=arg min⁡Ωf(Ω)=arg min⁡Ω(12||Z−ΩY||F2+γ4||ΩTΩ−I||F2).

The local minimum of the objective function (15) can be found by using a simple gradient descent method

(16) $$\begin{array}{c} \Omega^{t+1}=\Omega^{t}-\alpha \nabla f\left(\Omega \right), \end{array}$$

where *α* is a step size and ∇*f*(Ω) = −(**Z** − Ω**Y**)**Y** ^*T*^ + *γ*(ΩΩ^*T*^ − **I**)Ω. If the rows of Ω have different scales of norm, we cannot directly use the hard thresholding method to obtain **Z**. The phenomenon is called scaling ambiguity. Moreover, the constraint Ω^*T*^Ω = **I** can enforce Ω to be of full column rank but cannot avoid a subset of rows of Ω to be possibly zeros. Hence, we normalize the rows of Ω to prevent the scaling ambiguity and replace the zero rows, if any, by the normalized random vectors; that is,

(17) $$\begin{array}{c} {\hat{w}}_{i}=\{\begin{array}{cc} \frac{{\hat{w}}_{i}}{{||{\hat{w}}_{i}||}_{2}}, & {||{\hat{w}}_{i}||}_{2}\neq 0;\\ r, & \text{otherwise}, \end{array} \end{array}$$

where ${\hat{w}}_{i}$ is the *i*th row of $\hat{\Omega }$ and **r** is a normalized random vector. There may be other methods to prevent this problem, for example, by adding the normalization constraint to the objective function [7] or by adding the mutual coherence constraint on the rows of Ω to the objective function [12, 14]; however, this is out of the scope of our work. Although, after the row normalization, the columns of the dictionary are no longer close to the unit norm, the rank of $\hat{\Omega }$ will not be changed.

### 2.3. Convergence

In the step of updating **Z**, the algorithm to optimize the objective function (11) is analytical. Thus, the algorithm is guaranteed not to increase (11) and converges to a local minimum of (11) [21]. Furthermore, in the dictionary update step, Ω is updated by minimizing a fourth-order function, and thus a monotonic reduction in the cost function (15) is guaranteed.

Our algorithm (called orthogonality constrained ADL with iterative hard thresholding, OIHT-ADL) to learn analysis dictionary is summarized in Algorithm 1.

## 3. Computer Simulations

To validate the proposed algorithm, we perform two experiments. In the first experiment, we show the performance of the proposed algorithm for synthetic dictionary recovery problems. In the second experiment, we consider the natural image denoising problems. In these experiments, Ω_0_ ∈ *R* ^*P*×*M*^ is the initial dictionary in which each row is orthogonal to a random set of *M* − 1 training data and is also normalized [10]. For performance comparison, the AK-SVD [10], NAAOLA [8], and LOST [12] algorithms are used as baselines.

### 3.1. Experiments on Synthetic Data

**Figure 1:**
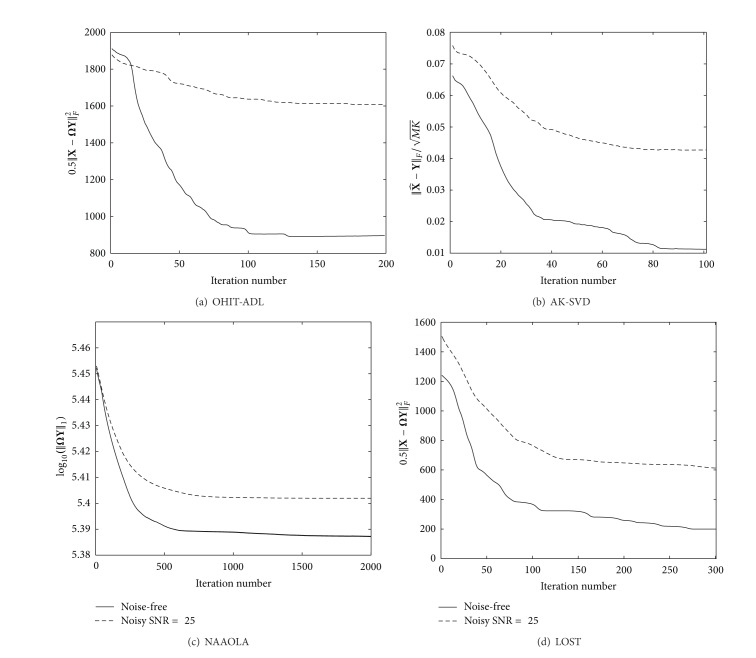
The convergence curves of the ADL algorithms for both the noise-free and noise case (where SNR = 25 dB).

**Figure 2:**
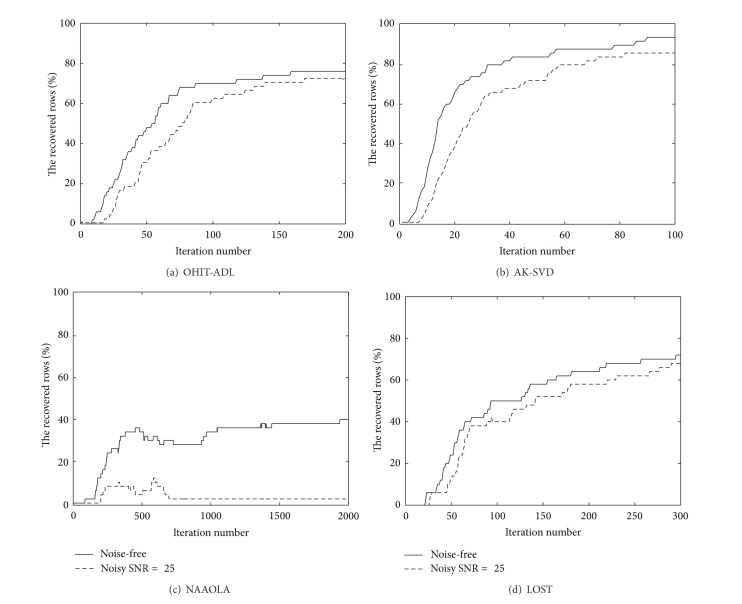
The recovery percentage curves of the ADL algorithms.

Following the work in [10], synthetic data are used to demonstrate the performance of the proposed algorithm in recovering an underlying dictionary Ω. In this experiment, we utilize the methods to recover a dictionary that is used to produce the set of training data. The convergence curves and the dictionary recovery percentage are used to show their performance. If $\min_{i}\left(1-|{\hat{w}}_{i}^{T}w_{j}|\right)<0.01$, a row **w**_*j*_^*T*^ in the true dictionary Ω is regarded as recovered, where ${{\hat{w}}_{i}}^{T}$ is an atom of the trained dictionary. Ω ∈ *R* ^50×25^ is generated with random Gaussian entries, and the dataset consists of *K* = 50000 signals each residing in a 4-dimensional subspace with both the noise-free setup and the noise setup (*σ* = 0.04, SNR = 25 dB). The parameters of the proposed algorithm are set empirically by experimental tests, and we choose the parameters as *λ* = 0.1, *γ* = 10, and *α* = 10^−4^ which give the best results in atom recovery rate. We have tested the parameters with different values in the ranges of 10^−2^ < *λ* < 10^2^, 10^−2^ < *γ* < 10^2^, and 10^−2^ < *α* < 10^−5^. For the AK-SVD algorithm, the dimension of the subspace is set to be *r* = 4 following the setting in [10]. For the NAAOLA algorithm, we set *λ* = 0.5 which is close to the default value *λ* = 0.3 in [8]. The parameters in the LOST algorithms are set as those in [11]; that is, *α* = 10^−4^, *λ* = *η* = *μ* = 10^5^, *p* = 20, and *s* = 29. The convergence curves of the error by the first term of (7) versus the iteration number are shown in Figure 1(a). It can be observed that the compared algorithms take different numbers of iterations to converge (the terms used to measure the rates of convergence are slightly different in these algorithms, because different cost functions have been used), which are approximately 200, 100, 1000, and 300 iterations for the OIHT-ADL, AK-SVD, NAAOLA, and LOST algorithms, respectively. Thus, the recovery percentages of these algorithms are measured at these different iteration numbers (to ensure that each algorithm converges to the stable solutions). It is observed from Figure 2 that 76%, 94%, 42%, and 72% of the rows in the true dictionary Ω are recovered for the noise-free case and 72%, 86%, 12%, and 68% for the noisy case, respectively. Note that the recovery rates for the LOST algorithm are obtained with random initialization. With the DCT and identity matrix initialization, the LOST algorithm can recover 74% and 72% rows of the true dictionary Ω in noise-free and noise cases, respectively, after 300 iterations. Although it may not be necessary to recover the true dictionary in practical applications, the use of atom recovery rate allows us to compare the proposed algorithm with the baseline ADL algorithms as it has been widely used in these works. The recovery rate by the NAAOLA algorithm is relatively low, mainly because it employs a uniformly normalized tight frame as a constraint on the dictionary and this constraint has limited the possible Ω to be learned. The AK-SVD algorithm performs the best in terms of the recovery rate, that is, taking fewer iterations to reach a similar recovery percentage. However, the running time in each iteration of the AK-SVD algorithm is significantly higher than that in our proposed OIHT-ADL algorithm. The total runtime of our algorithm for 200 iterations is about 825 and 832 seconds for the noise-free and noise case, respectively. In contrast, the AK-SVD algorithm takes about 11544 or 10948 seconds, respectively, for only 100 iterations (Computer OS: Windows 7, CPU: Intel Core i5-3210M @ 2.50 GHz, and RAM: 4 GB). This is because our algorithm does not need to estimate **X** in each iteration, as opposed to the AK-SVD algorithm.

### 3.2. Experiments on Natural Image Denoising

**Figure 3:**
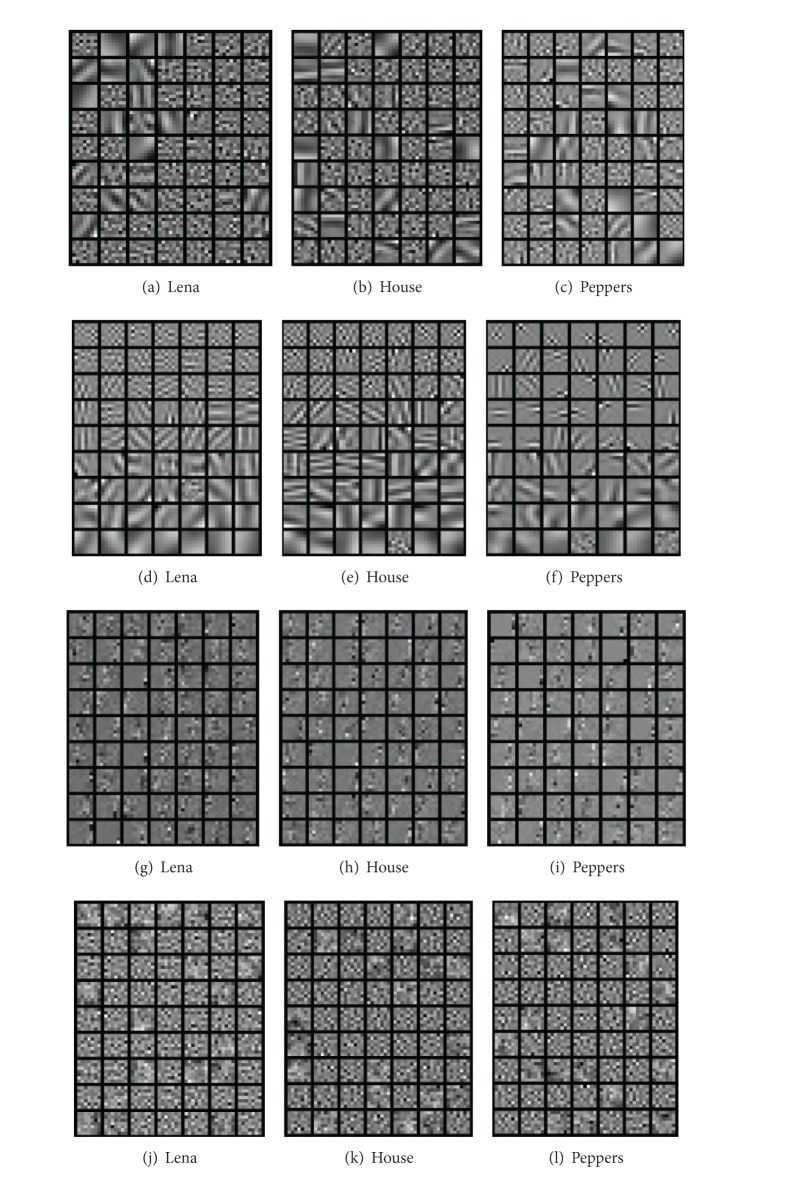
The learned dictionaries of size 63 × 49 by using the OIHT-ADL, AK-SVD, NAAOLA, and LOST algorithms on the three images with noise level *σ* = 5. The results of the OIHT-ADL are shown in the top row in (a) Lena, (b) House, and (c) Peppers, followed by the AK-SVD, NAAOLA, and LOST in the remaining rows.

In this experiment, the training set of 20,000 image patches, each of 7 × 7 pixels, obtained from three images (Lena, House, and Peppers) that are commonly used in denoising [10], has been used for learning the dictionaries, each of which corresponds to a particular noisy image. Noise with different level *σ*, varying from 5 to 20, is added to these image patches. The dictionary of size 63 × 49 is learned from the training data which are normalized to have zero mean. For fair comparison, the dictionary size is the same as that in [10]. The dictionary with a bigger size may lead to a sparser representation of the signal but may have a higher computational cost. Similar to Section 3.1, enough iterations are performed for the tested algorithms to converge. The parameters for the OIHT-ADL algorithm are set the same as those in Section 3.1, except that *λ* = 0.05. For the AK-SVD algorithm, the dimension of the subspace is set to be *r* = 7 following the setting in [10]. For the NAAOLA algorithm, we set *λ* = 3 (*σ* = 5), *λ* = 1 (*σ* = 10,15), and *λ* = 0.5 (*σ* = 20). For the LOST algorithm, the parameter values are set as *α* = 10^−11^, *λ* = *η* = *μ* = 10^5^, *p* = 20, and *s* = 21. The parameters for the NAAOLA algorithm and the LOST algorithm are chosen empirically. The examples of the learned dictionaries are shown from the top to the bottom row in Figure 3, where each atom is shown as a 7 × 7 pixel image patch. The atoms in the dictionaries learned by the AK-SVD algorithm are sorted by the number of training patches which are orthogonal to the corresponding atoms, and therefore the atoms learned by the AK-SVD algorithm appear to be more structured. Sorting is however not used in our proposed algorithm. Then we use the learned dictionary to denoise each overlapping patch extracted from the noisy image. Each patch is reconstructed individually and finally the entire image is formed by averaging over the overlapping regions. We use the OBG algorithm [10] to recover each patch image. For fair comparison, we do not use the specific image denoising method designed for the corresponding ADL algorithm such as the one in [12]. The denoising performance is evaluated by the peak signal to noise ratio (PSNR) defined as $\text{PSNR}=10\;\log_{10}(255^{2}\;\text{KM}/\sum_{i=1}^{K}\sum_{j=1}^{M}{\left({\hat{x}}_{ij}-x_{ij}\right)}^{2})$ (dB), where *x*_*ij*_ and ${\hat{x}}_{ij}$ are the *ij*th pixel value in noisy and denoised images, respectively. The results, averaged over 10 trials, are presented in Table 1, from which we can observe that the performance of the OIHT-ADL algorithm is generally better than that of the NAAOLA algorithm and the LOST algorithm. It is also better than that of the AK-SVD algorithm when the noise level is increased.

## 4. Conclusion

We have presented a novel optimization model and algorithm for ADL, where we learn the dictionary directly from the observed noisy data. We have also enforced the orthogonality constraint on the optimization criterion to remove the trivial solutions induced by a null dictionary matrix. The proposed method offers computational advantage over the AK-SVD algorithm and also provides an alternative to the LOST algorithm in avoiding the trivial solutions. The experiments performed have shown its competitive performance as compared to the three state-of-the-art algorithms, the AK-SVD, NAAOLA, and LOST algorithms, respectively. The proposed algorithm is easy to implement and computationally efficient.

## Figures and Tables

**Algorithm 1:**
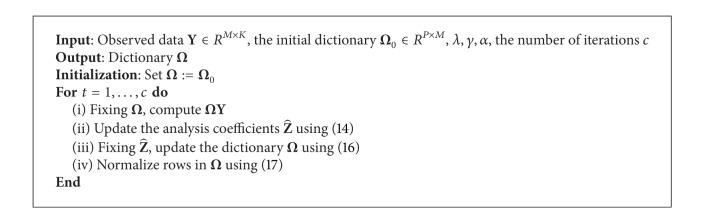
OIHT-ADL.

**Table 1:** Image denoising results (PSNR in dB).

σ	Noisy in dB	ADL method	Lena	House	Peppers
5	34.15	OIHT-ADL	38.26	38.08	37.70
		NAAOLA	37.36	37.04	35.93
		AK-SVD	38.44	39.20	37.93
		LOST	38.16	38.50	37.47

10	28.13	OIHT-ADL	35.02	34.79	33.89
		NAAOLA	32.73	32.61	31.12
		AK-SVD	34.85	35.32	33.82
		LOST	34.75	34.77	33.61

15	24.61	OIHT-ADL	33.17	33.05	31.69
		NAAOLA	30.87	30.82	29.22
		AK-SVD	32.59	32.98	31.28
		LOST	32.88	32.82	31.38

20	22.11	OIHT-ADL	31.83	31.71	30.13
		NAAOLA	29.65	29.22	27.40
		AK-SVD	31.38	31.51	29.76
		LOST	31.64	31.47	29.84
